# Relation between characteristics of carotid atherosclerotic plaques and brain white matter hyperintensities in asymptomatic patients

**DOI:** 10.1038/s41598-017-11216-x

**Published:** 2017-09-05

**Authors:** Enrico Ammirati, Francesco Moroni, Marco Magnoni, Maria A. Rocca, Roberta Messina, Nicoletta Anzalone, Costantino De Filippis, Isabella Scotti, Francesca Besana, Pietro Spagnolo, Ornella E. Rimoldi, Roberto Chiesa, Andrea Falini, Massimo Filippi, Paolo G. Camici

**Affiliations:** 1 Vita-Salute University and San Raffaele Hospital, Milan, Italy; 2 De Gasperis Cardio Center, Niguarda Hospital, Milan, Italy; 3 Vita-Salute University and Neuroimaging Research Unit, Institute of Experimental Neurology, and Division of Neuroscience, San Raffaele Scientific Institute, Milan, Italy; 4 Vita-Salute University and Department of Neuroradiology, San Raffaele Scientific Institute, Milan, Italy; 5 Department of Rheumatology, Istituto Ortopedico Gaetano Pini, Milan, Italy; 6 Cardiovascular Prevention Center, San Raffaele Institute, Milan, Italy; 7 CNR IBFM, Segrate, Italy

## Abstract

White matter hyperintensities (WMH) can be incidentally found in patients with carotid atherosclerosis on brain magnetic resonance imaging (MRI). We investigated the relationship between WMH and characteristics of carotid plaques in asymptomatic patients without indication for carotid revascularization. We prospectively screened 235 consecutive patients with carotid stenosis <70%. After excluding patients with confounding causes of cerebral damage, 67 asymptomatic patients underwent carotid computed tomography angiography (CTA), contrast-enhanced ultrasound and brain MRI. Number and quantitative measurement of volume of WMH were associated with history of resistant hypertension, degree of stenosis (Doppler) and presence of an ulcerated plaque at CTA (p < 0.05). At multivariate regression analysis, resistant hypertension was independently associated with both number and volume of WMH, presence of an ulcer with number of WMH and degree of stenosis with WMH volume (p < 0.05), although WMH were equally distributed in both hemispheres irrespectively of plaque side. In conclusion, in asymptomatic patients with carotid plaques <70%, a higher burden of WMHs is associated with history of resistant hypertension that could be the expression of microvascular damage. Stenosis severity and presence of plaque ulceration are also associated with WMH burden although their causative relation is not supported by the bilateral distribution of WMH.

## Introduction

The presence of a carotid plaque, especially if complicated or bearing signs of vulnerability, such as ulceration, or determining a high degree of stenosis can represent a major risk factor for the occurrence of ischemic stroke in asymptomatic subjects^1^. Previous studies demonstrated that individuals bearing carotid stenoses are subject to microvascular cerebral damage^2–6^. Such damage was shown to be associated with microembolic events^7, 8^, even if this issue remains controversial^9, 10^. Brain magnetic resonance imaging (MRI) allows identification and quantification of microvascular lesions. In accordance with recent neuroimaging standards, findings suggestive of microvascular damage include white matter hyperintensities (WMH) that are lesions in periventricular and deep white matter on T2 and/or fluid attenuated inversion recovery (FLAIR) sequences^11^. The relative importance and precise aetiology of these findings remain a subject of debate^12^. Previous studies have shown that individuals with asymptomatic plaques in the carotid arteries may have detectable microembolic signals on transcranial continuous-wave Doppler of cerebral blood vessels, which are associated with an increased risk of stroke^13^. A higher burden of microvascular lesions was also shown to correlate with risk of stroke^14–16^. Likewise, individuals suffering from atrial fibrillation (AF), a high risk for cardio-embolic events, have a higher number of microvascular lesions^17^.

So far, the management of carotid disease has relied primarily on stenosis severity^18^. It is possible that identification of specific plaque characteristics, in addition to traditional cardiovascular risk factors (CVRFs), might provide further insight on the propensity to subclinical brain damage. Computed tomography angiography (CTA) and contrast enhanced ultrasound (CEUS) allow fast and reproducible evaluation of plaque size and morphology, alongside with functional parameters^19, 20^. Plaque density and positive remodelling on CTA have been associated to histological features of plaque vulnerability^21, 22^. Similarly, compared to standard duplex evaluation, CEUS provides better definition of the plaque as well as information on intraplaque neovascularization^20, 23^.

Aim of this prospective study was the characterization of carotid plaques by means of multimodality imaging and their relation with WMH burden, in a cohort of asymptomatic subjects with at least one carotid plaque of intermediate severity without indication for carotid revascularization.

## Results

### Characteristics of the study population

Mean age of the study population was 69 ± 8 years and 43% were female subjects. Clinical characteristics, laboratory parameters and treatments at time of enrolment are summarized in Table 1.

**Table 1:** Characteristics of the study population.

**Demographic characteristics**	N = 67
Age, years	69 ± 8
Female, n(%)	29 (43)
**Cardiovascular Risk Factors**	
Family history of CAD, n(%)	23 (34)
Family history of stroke, n(%)	8 (12)
Systemic arterial hypertension, n(%)	51 (76)
Resistant hypertension, n(%)	12 (18)
Hypercholesterolemia, n(%)	47 (70)
Type 2 diabetes mellitus, n(%)	16 (24)
Current smoker, n(%)	13 (20)
Previous smoker, n(%)	29 (43)
Body mass index (kg/cm^2^)	25 ± 4
Framingham risk score (%)	13 (6–20)
High cardiovascular risk n(%)	36 (54)
**Cardiovascular History**	
Previous acute coronary syndrome, n(%)	7 (10)
**Clinical Characteristics**	
Heart rate, bpm	73 (67–80)
Systolic blood pressure, mmHg	130 (125–145)
Diastolic blood pressure, mmHg	80 (70–80)
**Laboratory Parameters**	
White blood cells, 10^9^/L	7.4 ± 1.7
Haemoglobin, g/dL	14 (13–15)
Platelets, 10^9^/L	205 (163–254)
Total cholesterol, mg/dL	175 (157–198)
LDL cholesterol, mg/dL	105 ± 33
HDL cholesterol, mg/dL	43 (38–50)
Triglyceridemia, mg/dL	129 (96–162)
Glycemia, mg/dL	99 (88–127)
Creatinine, mg/dL	0.85 (0.72–1.04)
eGFR, mL/min	72 (58–95)
**Medical Therapy**	
ACE inhibitors/ARBs, n(%)	41 (61)
β-blockers, n(%)	27 (40)
Calcium antagonists, n(%)	18 (27)
Diuretics, n (%)	15 (22)
Others vasodilators, n(%)	4 (6)
Number of anti-hypertensive agents	1 (0–2)
Statins, n(%)	40 (60)
Antiplatelet agent, n(%)	41 (61)

High cardiovascular risk score defined as Framingham risk score > 20%, and/or the presence of diabetes mellitus and/or manifest cardiovascular disease. CAD, coronary artery disease; HDL-C, high density lipoprotein cholesterol; LDL-C, low density lipoprotein cholesterol; eGFR, estimated glomerular filtration rate; ACE, angiotensin converting enzyme; ARBs, Angiotensin Receptor Blockers.

### Characteristics of carotid plaques

Table 2 summarizes baseline standard echographic, CEUS and CTA findings of the main plaque and of the atherosclerotic burden of the carotid arteries for each patient.

**Table 2:** Characteristics of carotid plaques.

Carotid Ultrasound Characteristics	N = 67
***Main plaque side***	
Left, n (%)	27 (40)
***Main plaque characteristics***	
Degree of stenosis (Doppler), n(%)	
<50%	40 (60)
50–70%	27 (40)
ECST (%)	51 ± 11
Grey scale, n (%)	
Lipid-rich plaques (I–II), n(%)	23 (34)
Fibro-calcific plaques (III–V), n(%)	44 (66)
CEUS^+^, n (%)*	29 (47)
*Atherosclerotic burden evaluation*	
CC-IMT (mm)	0.82 (0.75–1.01)
TPA (cm^2^)	0.80 (0.59–1.18)
Number of segments with plaque	4 (3–5)
Number of CEUS^+^ plaques*	1 (0–2)
Controlateral plaque > 40%, n(%)	38 (57)
**Carotid CT Angiography**	**N = 62**
***Main plaque characteristics***	
NASCET area, %	30 (10–40)
ECST area, %	55 (40–70)
Density, HU	302 (116–560)
Length, mm	16.9 (11.2–24.5)
Volume, mm^3^	125 (74–206)
Positive remodeling	0.5 (0.38–0.68)
Presence of ulcer, n(%)	9 (15)
Presence of luminal micro-calcification, n(%)	24 (39)
***Plaque burden***	
Total Plaque volume, mm^3^	300 (168–533)

ECST, European Carotid Surgery Trial; CEUS, contrast enhanced ultrasound; CC-IMT, common carotid intima media thickness; TPA = total plaque area; NASCET, North America Symptomatic Carotid Endarterectomy Trial; HU = Hounsfield Units. *Patients who underwent CEUS n = 62.

### Brain MRI findings

Median number of WMH was 28 per patient (interquartile range [IQR] 7–72), and WMH median volume was 761 mm^3^ (IQR 219–4053 mm^3^). No difference was found between right and left hemisphere in terms of number (p = 0.95) and volume (p = 0.74) of WMH. Results are summarized in Table 3.

**Table 3:** Brain magnetic resonance imaging findings.

BRAIN MRI	N = 67
**Total Burden of WMH**	
Number	28 (7–72)
Volume (mm^3^)	761 (219–4053)
**Right hemisphere Burden of WMH**	
Number	13 (4–39)
Volume (mm^3^)	368 (66–1490)
**Left hemisphere Burden of WMH**	
Number	14 (3–35)
Volume (mm^3^)	409 (84–1655)
**Burden of WMH - ipsilateral to the main plaque**	
Number	14 (3–36)
Volume (mm^3^)	375 (109–1607)
**Burden of WMH - contralateral to the main plaque**	
Number	16 (4–36)
Volume (mm^3^)	409 (67–1655)

Ipsilateral burden of WMH refers to the WMH located in the cerebral hemisphere on the same side of the main atherosclerosis lesion. Contralateral refers to the opposite hemisphere. WMH = white matter hyperintensities.

### CVRFs and total WMH burden

We explored the relation between patients’ demographics and their CVRFs and global burden of WMH detected on MRI. Interestingly, no association was found between age and WMH number (r = 0.06, p = 0.65) or volume (r = 0.08, p = 0.50). No gender difference was found in WMH number and volume. No significant associations were found (Supplementary Tables 1 and 2), with the exception that patients suffering from resistant hypertension had significantly more WMH (63 [17–122] vs. 28 [6–55], p = 0.04) and higher lesion volume (4166 [613–15265] vs. 644 [120–2075] mm^3^, p = 0.04) compared to normotensive subjects or hypertensive patients in whom blood pressure (BP) was well controlled by treatment. Hypertensive subjects did not have a significantly different burden of WMH in terms of number (30 [9–90] vs. 22 [3–33], p = 0.07) and volume (1003 [248–6525] vs. 535 [111–1816] mm^3^, p = 0.14).

### Carotid atherosclerosis and total WMH burden

We tested the association between the global atherosclerotic burden in the carotid arteries and the total amount of brain WMH. No correlation was found between common carotid intima-media thickness (CC-IMT) and WMH number (r = 0.00; p = 0.97) and volume (r = −0.01; p = 0.93). Total plaque area (TPA) did not correlate with WMH number (r = 0.14; p = 0.27) and volume (r = 0.17; p = 0.18). No correlation was found between the number of CEUS^+^ plaques and WMH number (r = −0.19; p = 0.15) and volume (r = −0.17; p = 0.18). Interestingly, the number of segments involved by the atherosclerotic process, a semi-quantitative marker of extensive, multisite atherosclerosis, was associated with higher volumes of WMH (r = 0.25, p = 0.04). The correlation of total number of segments involved by atherosclerosis with the number of WMH fell short of statistical significance (r = 0.23; p = 0.07) and the global volume of atherosclerosis in the carotid arteries measured by CTA did not correlate with WMH number (r = 0.04; p = 0.79) and volume (r = 0.01; p = 0.92).

### Main plaque characteristics and total WMH burden

**Figure 1:**
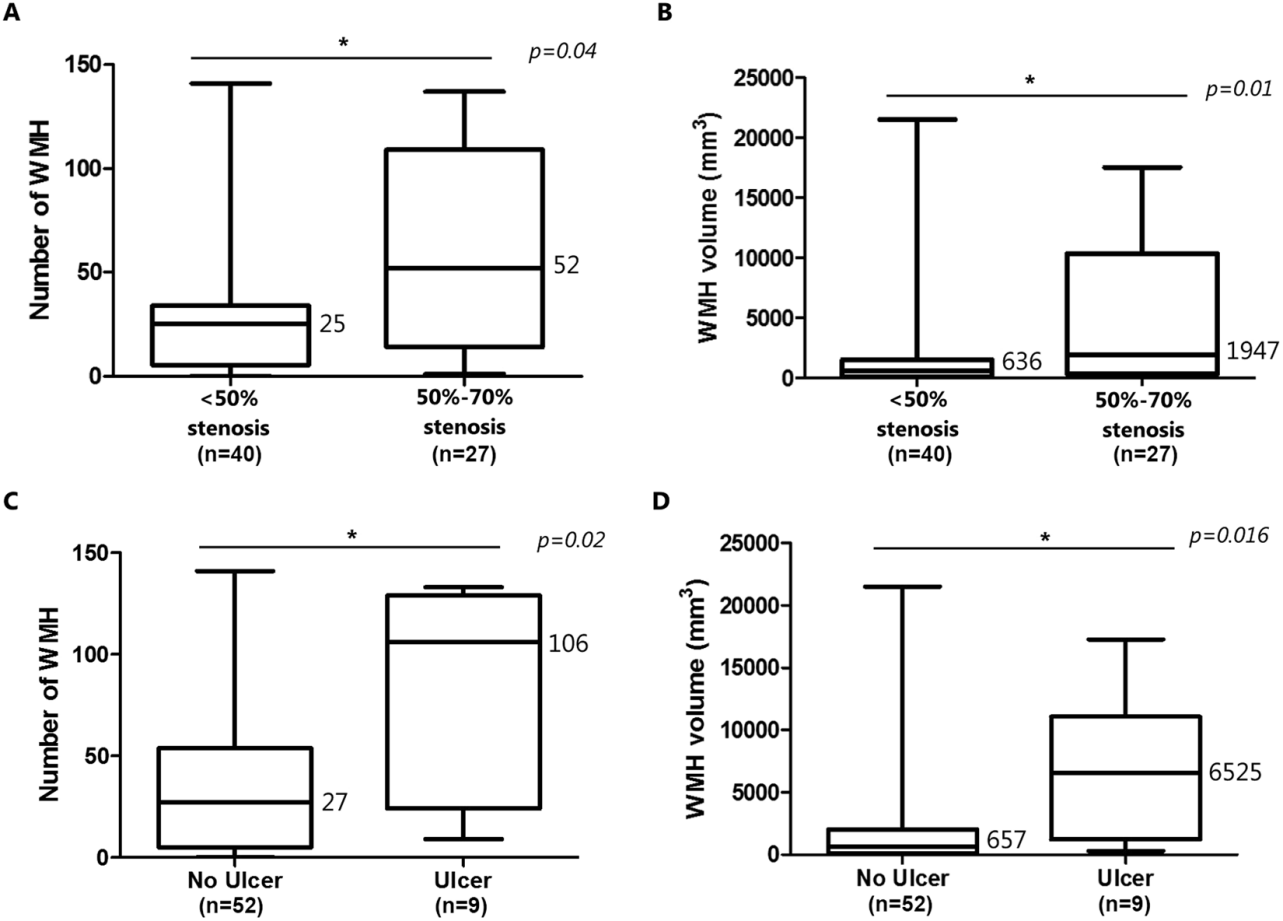
Box plot showing the significant associations between WMH and main plaque characteristics. Panel A shows the relation between the degree of stenosis and WMH number and volume. Panel B shows the relation between the presence of plaque ulcer on carotid CT angiography and WMH number and volume. Numbers indicate median value. WMH, white matter hyperintensities.

Patients with 50–70% stenosis measured with Doppler had a higher number of WMH compared with subjects bearing a stenosis <50% (Fig. 1A). On the other hand no association was found between WMH number and volume with the degree of stenosis measured using the European Carotid Surgery Trial (ECST) criteria on ultrasound images, nor with ECTS and North American Symptomatic Carotid Endarterectomy Trial (NASCET) area methods evaluated on CTA images, both when analyzed as continuous variable or dichotomized, i.e. <50 vs 50–70% (See Supplementary Table 3). Main plaque length and volume measured on CTA were not associated with WMH number or volume.

When analysis was focused on features of plaque vulnerability, the evidence of an ulcer on the main plaque, identified by CTA, was significantly associated with a higher burden of WMH in terms of both number and volume (Fig. 1B). Lipid-rich feature, plaque density, presence of plaque neovessels and luminal micro-calcifications or the degree of positive remodeling were not associated to WMH number or volume (Table 4 and Supplementary Table 4).

**Table 4:** Explored dichotomous associations between main plaque characteristics and WMH burden.

Characteristics	Number of WMH		Volume of WMH	
	Median	p	Median (mm^3^)	p
**Stenosis**				
• 50–70%	52	*0.04*	1947	0.01*
• <50%	25		636	
**Ulcer**				
• Yes	106	*0.02*	6525	0.016*
• No	27		657	
**Composition**				
• Lipid-rich	16	0.12	615	0.13
• Fibrocalcific	31		1048	
**CEUS+**				
• Yes	28	0.42	615	0.31
• No	30		1048	
**Microcalcifications**				
• Yes	17	0.75	693	0.64
• No	29		828	

WMH, white matter hyperintensities; CEUS, contrast enhanced ultrasound. *p < 0.05.

### Multivariate analysis to identify independent predictors of the global burden of WMH

We performed a multivariate regression analysis to identify independent predictors of WMH burden in terms of both number and volume. For what concerns WMH number, the variables included in the multivariate model were resistant hypertension, number of segments with plaque, degree of stenosis based on Doppler criteria, presence of plaque ulcer and age. We decided to include age in the model despite its lack of association with WMH number since age emerged as a predictor of WMH in previous studies^24, 25^. On multivariate analysis, resistant hypertension and presence of plaque ulcer were predictors of WMH number (model R^2^ = 0.28), while the association was borderline for the number of segments with plaque (p = 0.05). We included the same variables in the multivariate regression model for WMH volume. Resistant hypertension and degree of stenosis were the variables that remained significantly associated with WMH volume in the model (model R^2^ = 0.27). Table 5 reports the correlation coefficients and p-values for our model.

**Table 5:** Correlation coefficients and p-values of the multivariate regression model for the identification of predictors of white matter hyperintensities (WMH) number and volume.

**Variable**	Coefficients (95% CI)	p
**Predictors of WMH number**		
(Intercept)	−8.4 (−107.2; 90.5)	0.86
Age	−0.2 (−1.6; 1.2)	0.78
Resistant hypertension	**28.4 (0.6; 56.2)**	0.04*
Number of segments with plaque	7.0 (−0.2; 14.2)	0.05
Presence of ulcer	**35.5 (5.3; 65.7)**	0.02*
Degree of stenosis (Doppler)	18.1 (−3.5; 39.6)	0.09
**Predictors of WMH volume**		
(Intercept)	−3351.2 (−15744.8; 9042.5)	0.59
Age	−9.8 (−195.9; 156.4)	0.82
Resistant hypertension	**3942.3 (452.6; 7431.9)**	0.02*
Number of segments with plaque	743.4 (−154.6; 1641.4)	0.10
Presence of ulcer	3088.4 (−701.6; 6879.2)	0.10
Degree of stenosis (Doppler)	**2871.2 (162.8; 5579.5)**	0.03*

95% CI = 95% confidence interval. *p < 0.05.

### Hemispheric differences in WMH burden

No difference in WMH number and volume was found between the hemisphere ipsilateral to the main plaque and the contralateral one (14 vs. 16, p = 0.40 and 375 vs. 409 mm^3^, p = 0.20 respectively). No hemispheric difference was found upon the exclusion of subjects having a contralateral stenosis > 40% in diameter (13 ipsilateral vs. 10 contralateral, p = 0.95 and 368 mm^3^ ipsilateral vs. 284 mm^3^ contralateral, p = 0.76 respectively). Then, we tested whether features of the main plaque that were found to be increased in patients with higher WMH burden were associated with increased lesions on the side of the main plaque. No hemispheric difference in WMH was found in subjects with a degree of stenosis of 50–70% according to Doppler criteria (24 ipsilateral vs. 28 contralateral, p = 0.72, 909 mm^3^ ipsilateral vs. 805 mm^3^ contralateral, p = 0.94 respectively). No hemispheric difference in WMH number and volume was found among patients with evidence of main plaque ulceration (57 ipsilateral vs. 49 contralateral, p = 0.73 and 3178 mm^3^ ipsilateral vs. 2972 mm^3^ contralateral, p = 0.80 respectively).

## Discussion

In asymptomatic patients with intermediate carotid plaque, the history of resistant hypertension was independently associated with both number and volume of total WMH burden. Two features of the main plaque, i.e. the presence of an ulcer on CTA and the degree of stenosis based on Doppler criteria (50–70%), further emerged as independent predictors of the total number and volume of WMH burden.

Our data confirm that hypertension, in particular its resistant form, is strongly associated with WMH^12^. Hypertension is indeed a key pathogenic factor for cerebral small vessel disease, of which WMH burden is a cardinal manifestation. The chronic leakage of plasma protein and fluid, secondary to hemodynamic damage to the blood brain barrier, is believed to contribute to tissue damage observed in WMH^12^. We observed that age in this population had no association with WMH number, even if emerged as a predictor of WMH in previous studies^27, 28^. A potential explanation is that our population had a narrow age distribution (between 60 and 80 years) thus reducing the potential effect of age on brain WMH comparing to previous studies that included healthy subjects from general population with a by far larger age span. Our data show that the degree of stenosis estimated with Doppler criteria, but not using morphological parameters, was associated with a higher burden of WMH. The finding that Doppler velocity measurements were associated with the burden of WMH confirms previous observations from large population-based studies^8, 10^.

This is the first study to systematically assess carotid stenosis both with functional and morphological criteria, and to relate it to subclinical cerebral damage, and thus, to identify the discrepancy between the two measures. In our opinion, this apparent contradiction can be explained by the substantial difference between measuring a morphological and a functional parameter. Doppler evidence of flow acceleration, which is at the base of velocimetric estimates of stenosis, recapitulates not only the degree of stenosis, but also the length of the plaque, the stiffness of the arterial wall and its compliance^26^, and eventually high cerebral resistance^27^. Thus, the identified acceleration may not be interpreted merely as the result of the narrowing of the lumen, but may be considered broadly as being the expression of a more general compromise of the carotid and cerebral circulation.

For the first time we demonstrated an association between the total number of WMH and evidence of main plaque ulcer on carotid CTA, that is recognized as a potential marker of plaque vulnerability^19, 20^. While this finding may point at a direct pathogenic role of recurrent microembolism from unstable plaque in the occurrence of WMH^13^, our results seem to refute this hypothesis. Indeed, no difference in WMH burden was evident between the hemisphere subtended by the main plaque and the contralateral neither in the entire study population nor in the group of subjects with evidence of ulcer in the main plaque.

Asymmetry in cerebral involvement by small vessel disease in subjects with carotid atherosclerosis would strongly support an etiologic role for the carotid plaque. Indeed while a recent retrospective study has linked carotid atherosclerosis to the ipsilateral development of cortical silent brain infarction (but not of WMH)^28^, other studies failed to demonstrate an association between carotid plaque and ipsilateral WMH^3, 9, 29^. Thus, we believe that the presence of ulcer in the plaque may distinguish subjects with a globally more aggressive atherosclerotic disease rather than characterizing the single plaque as vulnerable. The presence of an ulcerated carotid artery plaque may mark a vulnerable patient as opposed to a vulnerable plaque^30^.

It is worth noting that R^2^ for our multivariate model for WMH number and volume were 0.28 and 0.27 respectively. This means CVRFs and carotid plaque characteristic combined failed to account for 72–73% of total WMH burden variance. This is in line with recently published findings on 882 subjects from the *Lothian Birth Cohort 1936*, in which the combination of multiple CVRFs could explain only a minimal part of WMH burden variance^31^. In fact, while currently WMH are considered ischemic in nature, a recent multi-ethnic genome wide association study demonstrated that the variance of WMH may be associated with loci related to neural inflammation and apoptosis regulation in glial cells^32^. Furthermore, the confounding effect of potential sources of subclinical brain damage other than carotid atherosclerosis must be taken in account, such as silent paroxysmal AF.

### Study limitations

The main limitation of the present work relates to its relatively small sample size, which prevents a ready generalization of its findings and may result in a low statistical power of the study. However, the effect of the small number of individuals on the power of the study may be mitigated by the fact that this prospective study is the first to apply extremely strict inclusion and exclusion criteria in order to minimize the potential confounders.

Another limitation relates to the field of WMH research per se rather than to our study in particular. While the clinical relevance of WMH is well established^33^, and the identification of these lesions follows internationally recognized neuro-imaging standards^11^, the quantification of cerebral involvement of WMH is still a matter of debate. Indeed, most of previously published studies employed semi-quantitative visual scores, including Fazekas scale or Wahlund score^11^. Volumetric quantification of WMH is currently advocated as a mean of improving reproducibility, but no clinically significant volumetric cut offs in terms of WMH burden have been identified to date^11^. In line with these indications, this is the first study that assesses the relation between features of carotid plaques and quantitative measurement of WMH burden representing a step forward compared to previous studies.

We also acknowledge that the main outcome of our study is an intermediate outcome, which hampers the ready translation of our findings to daily clinical practice in the fields of stroke and dementia prevention. However, WMH were shown to be strong predictors of both^33^, thus we deem our results as promising and relevant in clarifying the complex relation between brain and cardiovascular system.

## Conclusions

Resistant hypertension and carotid atherosclerosis characteristics, in particular main plaque stenosis estimated with Doppler criteria and presence of an ulcer on the main plaque, were associated with an increased burden of WMH. No plaque feature was associated with an increased ipsilateral burden of WMH. Based on the results of our study, albeit preliminary and obtained in a small, but carefully selected population, we believe that carotid atherosclerosis may not have a direct causal role in the development of WMH. Indeed, however, considering the indirect associations of carotid plaque features and WMH, a thorough evaluation of carotid atherosclerosis beyond the degree of stenosis may help the clinician identifying those patients in which a higher burden on incompletely controlled risk factors is damaging on the brain. This hypothesis must be supported by longitudinal data, which is the future objective of the IMPLAC study.

## Methods

### Study design and population

**Figure 2:**
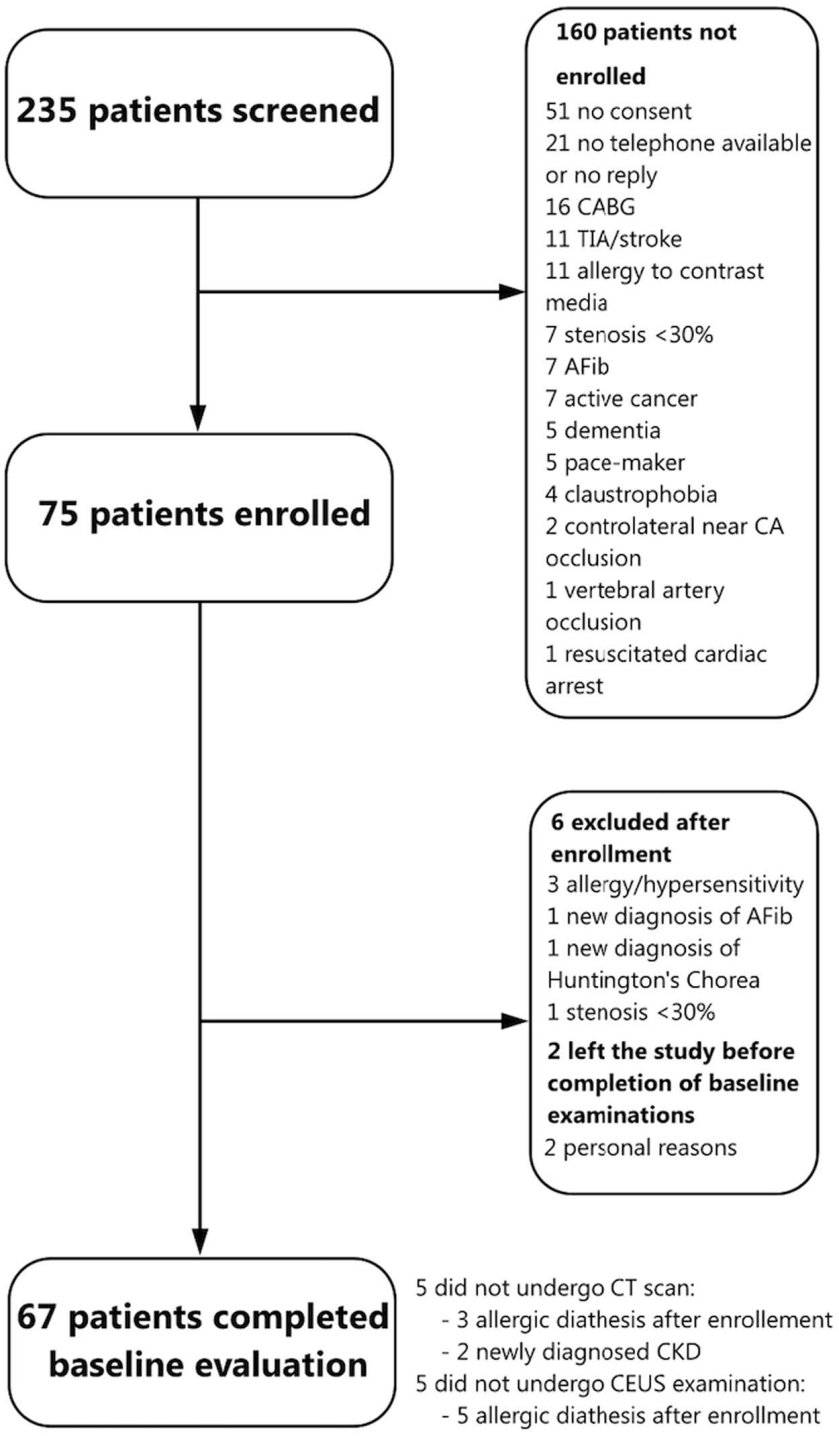
Patient selection diagram. CABG, coronary artery bypass grafting; TIA, transient ischemic attack; AFib, atrial fibrillation; CA, carotid artery; CT, computed tomography; CKD, chronic kidney disorder; CEUS, contrast enhanced ultrasound.

This population represents the baseline cohort of a prospective study to assess plaque features and CVRFs in relation to WMH progression (Imaging della PLAcca Carotidea, IMPLAC study). The study was approved by the Ethics Committee of the San Raffaele Hospital (date of approval January, 30^th^ 2012, protocol name IMPLAC) and all expeiments were performed in accordance with relevant guidelines and regulations. All subjects provided informed consent to take part in the study, in compliance with the Declaration of Helsinki and Good Clinical Practice. We enrolled consecutive, asymptomatic subjects with carotid stenosis <70% according to Doppler velocity measurement and with a focal protruding plaque that caused at least a 30% stenosis using ECST method on B-mode ultrasound images^34^. Therefore, based on these findings the patients were not deemed eligible for carotid revascularization by the referring specialist. Stringent exclusion criteria were set, in order to avoid potential confounding causes of cerebral damage, including: age <18 or >85 years, pregnancy, history of allergic diathesis, previous stroke or transitory ischemic attack, previous carotid endarterectomy (CEA) or stenting, vasculitis, history of alcohol or drug abuse, life expectancy of <18 months, presence of cognitive impairment preventing the patient from providing informed consent, history or current AF or previous cardiac surgery as potential confounding causes of cerebral ischemic damage (Supplementary Table 5 for details). An ECG monitoring for the all duration of the CEUS and a fast transthoracic echocardiogram examination were performed in all patients to further limit the impact of potential cardio-embolic events. Between April 2012 and November 2015, we screened 235 patients referred to Ospedale San Raffaele Neurophysiology and Vascular Surgery outpatient services for routine ultrasound evaluation of the carotid arteries. All asymptomatic subjects had been referred by their primary care physician for carotid ultrasound as a part of the cardiovascular risk assessment. The largest proportion of patients underwent carotid ultrasound examination for the first time due to presence of multiple CVRFs or a history of coronary artery disease. Other patients had a known intermediate carotid plaque and they underwent this exam as follow up. A complete overview of the subject selection process and enrolment is presented in Supplementary expanded methods and Fig. 2. All patients underwent carotid ultrasound imaging, CEUS, carotid CT and brain MRI. All these examinations were performed within 3 weeks, to minimize the bias introduced by possible disease progression.

### Conventional carotid Ultrasound Imaging

All patients underwent bilateral Duplex ultrasound evaluation of the carotid arteries. Examinations were performed by a single expert sonographer (M.M.) using a dedicated ultrasonography equipment (Logiq S8, GE Healthcare, UK) with a 7-MHz linear probe (7 L, GE). For details of ultrasound-derived parameters see Supplementary methods.

### Contrast enhanced ultrasound of the carotid arteries

CEUS examination was performed using the same dedicated equipment. Preset constrast-specific modality (pulse inversion) was employed, adjusting image settings in order to maximize contrast signal visualization and using low mechanical index (0.08–0.12). Five ml of sodium hexafluoride (SonoVue, Bracco Imaging, Italy) were diluted 1:3 in saline. A 3 ml bolus was injected through a peripheral vein and then flushed with 5 ml saline. We did not perform CEUS examination in 5 patients, due to recent history of allergic reactions to drugs, which happened after the enrolment. Plaque neovascularization was assessed offline by 3 independent operators (M.M., F.M. and E.A.) as previously described^23, 35^. Each lesion was graded as CEUS^−^ or CEUS^+^ depending on absence or presence of neovascularization signal, respectively. CEUS^+^ plaques were defined as plaques in which contrast bubbles, identified as moving bright spots, reached the plaque core or in which contrast signal was seen throughout the entire lesion^36^. For each patient, the total number of neovascularized plaques was recorded as a semi-quantitative index of neoangiogenetic activity. No adverse reactions to SonoVue were reported. Inter-observer reproducibility for the detection of CEUS^+^ plaques was 95%. Any discordance amongst the operators was resolved by consensus. Intra-observer reproducibility was determined re-assessing a subset of 15 examinations, presented in a random order, after a week from the first analysis and resulted in 98% agreement.

### Computed Tomography Angiography

All patients were examined with a 64-slice CT scanner (VCT Lightspeed, GE Healthcare, USA). Fifty ml of non-ionic, iso-osmolar contrast material (Iodixanol, 320 mg of iodine per millilitre, Visipaque 320; GE Healthcare, USA) prepared at 37 °C was injected into an antecubital vein through a 20 or 18-gauge catheter at a rate of 5 ml/s, followed by the injection 50 ml of saline. Contrast administration was performed using a dual-shot injector (Nemoto Kyorindo, Japan). The helical acquisition was initiated after the bolus reached the aortic arch using a visual bolus tracking. Data were acquired from the aortic arch to the vertex. The section thickness was 0.625 mm, pitch was 0.9 mm/rot, field of view was large. Tube voltage and current were 120 kV and 250 mA, respectively. The average absorbed radiation dose per patient was approximately 0.68 mSv. Images were reconstructed with a slice thickness of 0.625 mm using a soft-tissue convolution kernel, transferred to an external workstation (AW 4.5, GE Healthcare) for post-processing image analysis and reviewed by two experienced radiologists (F.B. and P.S.), blinded to other imaging data and clinical information. Further details on analyzed parameters of the plaque are presented in Supplementary methods.

### Brain magnetic resonance imaging

In all subjects, the following sequences of the brain were collected during a single session using a 1.5 Tesla scanner (ACHIEVA Philips Medical Systems): (a) two-dimensional (2D) fluid-attenuated inversion recovery (FLAIR, repetition time [TR]/echo time [TE] = 11000/140 ms, inversion time [TI] = 2800 ms, echo train length [ETL] = 47, flip angle [FA] = 90°, matrix size = 384 × 253, field of view [FOV] = 230 × 230 mm^2^, 44 axial 3 mm-thick slices, number of excitations [NEX] = 3, SENSE = 1.5, acquisition time [TA] = 7′20″); (b) 2D T2-weighted turbo spin-echo (TSE) (TR/TE = 8897/100 ms, ETL = 15; flip angle [FA] = 90°, matrix size = 384 × 242, FOV = 230 × 230 mm^2^, 44 axial 3mm-thick slices, NEX = 3, SENSE = 1.5, TA = 4:9) and (c) 3D T1-weighted transient field echo (TFE) (TR/TE = 7/3.2 ms, TI = 900 ms, FA = 8°, matrix size = 256 × 256, FOV = 256 × 256 mm^2^, 192 sagittal 1.2 mm-thick slices, NEX = 1, SENSE = 2, TA = 6′47″).

**Figure 3:**
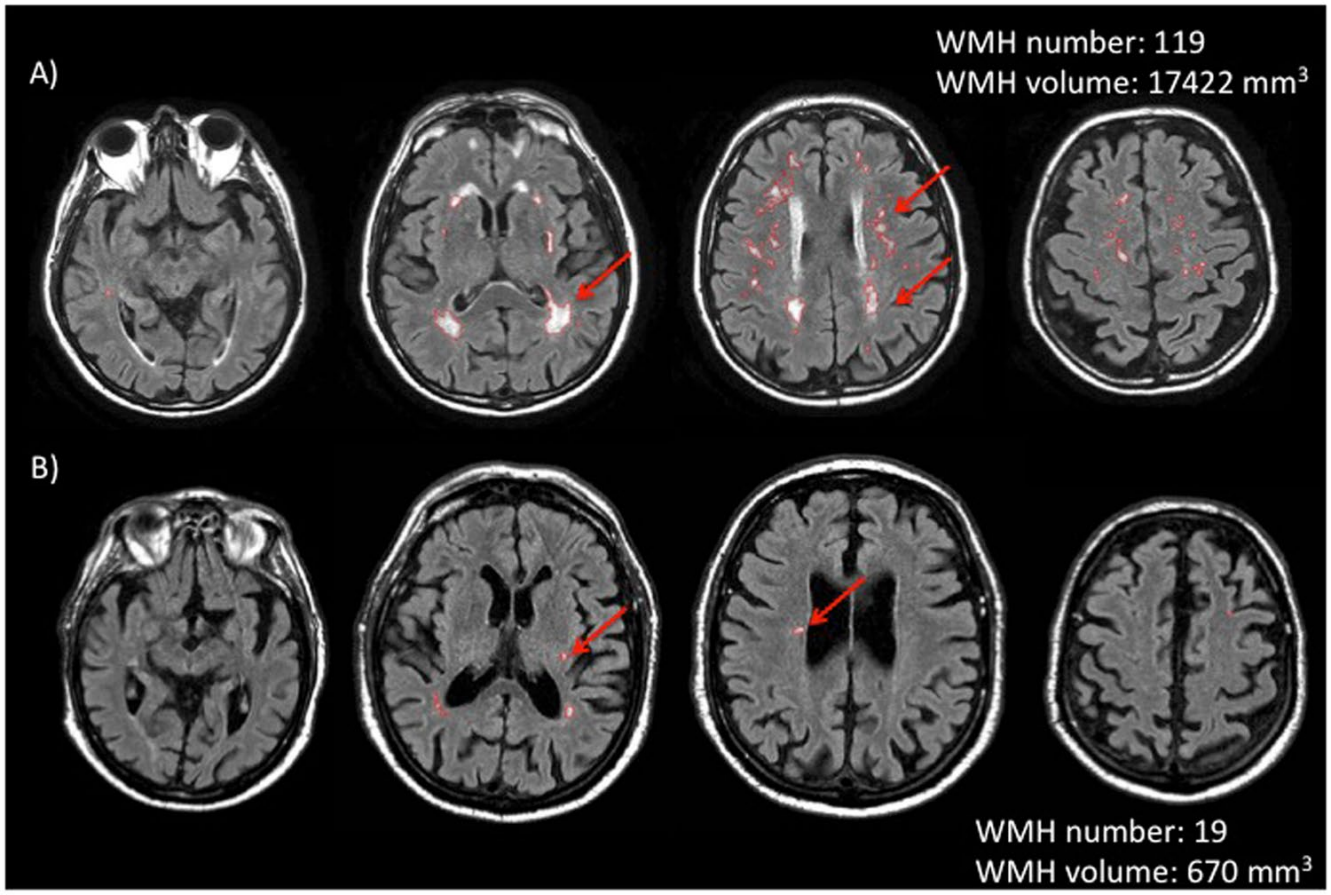
Two representative cases of brain Magnetic Resonance Imaging analysis. Top row: images from a subject with a high number/volume of white matter hyperintensities (WMH) are shown. Bottom row: scans of a subject with a low number/volume of WMH. In both cases, WMH are countered in red, by using semi-automated software. Arrows underlines some of the WMH.

FLAIR-hyperintense lesions, i.e. WMH, were identified by consensus by two experienced observers blinded to carotid arteries CT and ultrasound evaluation (R.M. and C.D.F.). FLAIR lesion volume (LV) was quantified using a local thresholding segmentation technique (Jim 6, Xinapse Systems, West Bergholt, UK; http://www.xinapse.com/)^37, 38^. FLAIR lesion volume and number was reported for the whole brain and for each hemisphere separately. While earlier reports suggested that deep and periventricular WMH have different pathogenesis, more recent studies have led to the hypothesis that WMH are globally the expression of a common aetiology^6, 12^. Thus, we have decided to evaluate WMH volume and number globally, without distinction between deep and periventricular. Figure 3 shows brain MRI analysis of two representative patients.

### Statistical analysis

All continuous variables were tested for normality using Shapiro-Wilk normality test and are expressed as mean ± standard deviation (SD) or median [interquartile range (Q1-Q3)] as appropriate. Categorical variables are summarized as absolute frequency (percentage). Group differences were tested using unpaired t test, Mann-Whitney U test or Wilcoxon matched pair signed rank test. Spearman’s rank correlation coefficient was used to assess statistical dependence. Association between categorical variables was tested using Chi-squared test or Fisher’s exact test as appropriate. A p < 0.05 was considered significant. A multivariate regression analysis was then performed to identify factors that independently influence the burden of WMH in terms of number and volume. All variables associating to WMH burden with a p < 0.10 were included in the multivariable model. We decided a priori to include age a priori age in the model since age emerged as a robust predictor of WMH in previous studies^24, 25^. In case of two variables that were not independent of one another, in the multivariate we have included the variable with the highest significance, as in the case of resistant hypertension and hypertension. All statistical analyses were performed using GraphPad Prism 5 (GraphPad Software Inc., La Jolla, USA), IBM SPSS Statistics 20 (IBM, Armonk, USA) or R v3.1.2.

## Electronic supplementary material

Supplemental materials
