# Supplementary material for: Relation between characteristics of carotid atherosclerotic plaques and brain white matter hyperintensities in asymptomatic patients
Source: Sci Rep. 2017 Sep 5;7:10559. doi: 10.1038/s41598-017-11216-x (PMC5585357; doi:10.1038/s41598-017-11216-x)
Supplement: Supplementary file 1 — Supplemental materials [file 41598_2017_11216_MOESM1_ESM.doc]

Relation between characteristics of carotid atherosclerotic plaques and brain white matter hyperintensities in asymptomatic patients

Ammirati et al. Carotid plaques and WMHs in asymptomatic patients

Enrico Ammirati^1,2^, MD, PhD, Francesco Moroni^1^, MD, Marco Magnoni^1^, MD, Maria A. Rocca^3^, MD, Roberta Messina^3^, MD, Nicoletta Anzalone^4^, MD, Costantino De Filippis^4^, MD, Isabella Scotti^5^, MD, Francesca Besana^6^, MD, Pietro Spagnolo^6^, MD, Ornella E. Rimoldi^7^, MD, Roberto Chiesa^1^, MD, Andrea Falini^4^, MD, Massimo Filippi^3^, MD, and Paolo G. Camici^1^, MD.

1 Vita-Salute University and San Raffaele Hospital, Milan, Italy

2 De Gasperis Cardio Center, Niguarda Hospital, Milan, Italy

3 Vita-Salute University and Neuroimaging Research Unit, Institute of Experimental Neurology, and Division of Neuroscience, San Raffaele Scientific Institute, Milan, Italy

4 Vita-Salute University and Department of Neuroradiology, San Raffaele Scientific Institute, Milan, Italy.

5 Department of Rheumatology, Istituto Ortopedico Gaetano Pini, Milan, Italy

6 Cardiovascular Prevention Center, San Raffaele Institute, Milan, Italy.

7 CNR IBFM, Segrate, Italy

Subject terms: Peripheral vascular disease, atherosclerosis, ischemic stroke, magnetic resonance imaging (MRI), ultrasound.

DATA SUPPLEMENTS

EXPANDED METHODS AND RESULTS

Study design and population

At enrolment, all subjects underwent clinical evaluation including careful medical history, and physical examination. The history was focused on the identification of cardiovascular risk factors (CVRFs) and of current treatment. For each patient, glycaemia, total cholesterol, high-density lipoprotein cholesterol (HDL-C), low-density lipoprotein cholesterol (LDL-C) and triglycerides were measured using a colorimetric method using the Cobas Mira Plus analyzer (Horiba, ABX, France).

A patient was defined as suffering from hypertension in compliance with the definition of the European Society of Cardiology (ESC)/European Society of Hypertension, i.e. systolic blood pressure (BP) ≥140mmHg or diastolic BP ≥90mmHg.[^1^](#_ENREF_1) A clinical history of hypertension with current BP medication was also considered diagnostic. A subject was considered as suffering from resistant hypertension in the presence of uncontrolled hypertension or a history of poorly controlled BP and a current BP treatment with at least three drugs including a diuretic.[^2^](#_ENREF_2) A fasting blood glucose >126 mg/dL, a glycaemia >200 mg/dL two hours after an oral glucose load of 75g, HbA1C levels over 6.5% or current treatment with oral anti-diabetic agents or insulin were taken into account for the diagnosis of diabetes.[^3^](#_ENREF_3) Hypercholesterolemia was defined according to ESC guidelines: total cholesterol ≥200 mg/dL and /or LDL-C ≥120 mg/dL.[^4^](#_ENREF_4) Current lipid lowering therapy was also taken into consideration for the definition of hypercholesterolemia. To evaluate the cardiovascular risk profile of our patients, we employed the Framingham Risk Score (FRS) to estimate the probability of a first acute myocardial infarction at 10 years.[^5^](#_ENREF_5) The variables taken into consideration are age, systolic BP, current smoke, BP medication, total cholesterol and HDL-C. A patient with a FRS>20% is considered at high risk. The presence of diabetes mellitus or manifest cardiovascular disease confer high risk independently from calculated FRS.[^5^](#_ENREF_5)

Conventional carotid Ultrasound Imaging

The patient was positioned supine, with the head tilted 45° toward the side opposite to the one being imaged. A transverse sweep was recorded from the lower neck to the common carotid artery (CCA) bifurcation into the external carotid artery (ECA) and internal carotid artery (ICA) in order to identify atherosclerotic lesions, defined as focal lesions exceeding 2 mm in thickness. Each of the segments was identified according to the Rotterdam Study criteria: the 15 mm caudal to the bifurcation was defined as carotid bulb, while cranially we identified ICA and ECA. Longitudinal images were then recorded using anterior, posterior and lateral (45°) views.

Common carotid intima-media thickness (CC-IMT) measurement was performed bilaterally in lateral projection images of the CCA in a semi-automated manner. Briefly, the measures performed on 350 consecutive points taken from approximately 10 mm proximal from the bulb were averaged. We carefully avoided inclusion of CCA atherosclerotic plaques in the measurement of CC-IMT. We recorded the highest mean CC-IMT for each patient.

Doppler velocity measurements were made on longitudinal views at the site of any identifiable lesions within CCA, carotid bulb or ICA. The degree of stenosis was evaluated by velocimetric criteria according the Society of Radiologists in Ultrasound Consensus Conference.[^8^](#_ENREF_8) The plaque determining the highest stenosis was considered the main lesion. For the main lesion, we calculated the degree of stenosis according to the European Carotid Surgery Trial (ECST) criteria, as previously described.[^9^](#_ENREF_9) Briefly, the narrowest diameter and the estimated normal diameter of the artery at the site of the main plaque were measured, and the degree of stenosis was calculated as [1-(narrowest diameter/estimated normal diameter)]%.

For each patient, Total Plaque Area (TPA) was measured off-line as previously described.[^10^](#_ENREF_10) Briefly, two independent operators (M.M. and F.M) measured the two dimensional area of each identifiable lesion by tracking around the lesion perimeter. The sum of all lesions areas was taken as TPA. The number of segments, CCA, bulb, ICA and ECA bilaterally, involved by atherosclerosis was also registered as an indicator of the extension of the disease.

Finally, each plaque was classified according to its echogenicity, as previously described.[^11^](#_ENREF_11) Briefly, plaques were considered class I if they were uniformly hypoechoic, class II if they were heterogeneous, mainly hypo-echoic, class III if they were heterogeneous, mainly hyperechoic, class IV if they were uniformly iso-hyper-echoic or class V if they were hyperechoic and displayed posterior acoustic shadow. For subsequent analysis, class I and II were considered a single category, namely lipid-rich plaque, while classes III to V were considered together as fibrocalcific plaques.

## **Computed Tomography Angiography**

The degree of stenosis in CCA, carotid bulb and ICA were analyzed according to North American Symptomatic Carotid Endarterectomy Trial (NASCET) and ECST criteria. Briefly, for what concerns the NASCET method, the narrowest luminal area at the site of the stenosis and the area of normal artery distal to the plaque were measured, and the degree of stenosis was calculated as [1-(narrowest area/normal area)]. For the ECST, the method is analogous as for what is described above for duplex imaging, but areas were used instead of diameters. The axial data and multiplanar reconstruction (MPR) were used to determine the grade of stenosis, to calculate the plaque volume and the composition of the plaque, defined on the basis of plaque density in Hounsfield unit (HU). Five patients did not undergo CT angiography (CTA) due to specific contraindications to the administration of iodinated contrast material, in particular 3 subjects had a recent history of allergic reactions to drugs while 2 subjects were diagnosed chronic kidney disease after enrolment in the study.

ADDITIONAL TABLES

SUPPLEMENTARY TABLE 1. Explored correlations between continuous clinical variables and number and volume of white matter hyperintensities (WMH) respectively.

SBP, systolic blood pressure; DBP, diastolic blood pressure; eGFR, estimated glomerular filtration rate; BMI, body mass index; HDL-C, high density lipoprotein cholesterol; LDL-C, low density lipoprotein cholesterol.

|  | Number of WMH | | Volume of WMH | |
| --- | --- | --- | --- | --- |
| Clinical parameter | r | p | r | p |
| Age | 0.057 | 0.65 | 0.084 | 0.50 |
| SBP | -0.047 | 0.72 | -0.004 | 0.97 |
| DBP | 0.033 | 0.80 | 0.077 | 0.55 |
| eGFR | -0.165 | 0.21 | -0.102 | 0.44 |
| BMI | 0.052 | 0.67 | 0.056 | 0.65 |
| Total cholesterol | 0.028 | 0.83 | 0.037 | 0.78 |
| HD-C | -0.05 | 0.72 | -0.09 | 0.50 |
| LDL-C | -0.04 | 0.78 | -0.017 | 0.90 |
|  | | | | |

SUPPLEMENTARY TABLE 2. Associations between dichotomous clinical variables and global white matter hyperintensities (WMH) burden.

CAD, coronary artery disease; T2DM, type 2 diabetes mellitus; HTN, hypertension; CVD, cardiovascular disease.

|  | Number of WMH | | Volume of WMH | |
| --- | --- | --- | --- | --- |
| Clinical parameter | Medians | p | Medians (mm^3^) | p |
| CAD   - Yes - No | 32  28 | 0.83 | 1626  670 | 0.55 |
| T2DM   - Yes - No | 22  29 | 0.30 | 499  1092 | 0.29 |
| Smoker   - Yes - No | 30  20 | 0.83 | 973  722 | 0.98 |
| HTN   - Yes - No | 30  22 | 0.07 | 1003  535 | 0.14 |
| Resistant HTN   - Yes - No | 63  28 | *0.04* | 4166  644 | *0.04* |
| High CVD risk   - Yes - No | 28  28 | 0.75 | 664  973 | 0.91 |
|  | | | | |

SUPPLEMENTARY TABLE 3. Carotid stenosis degree based on European Carotid Surgery Trial (ECST) based on echographic and CT images in relation to extent and volume of white matter hyperintensities (WMH).

| ECST B-mode Ultrasound | | | |
| --- | --- | --- | --- |
| WMH | <50 | 50-70 | P |
| Number | 28 (8-34) | 30 (6-89) | 0.54 |
| Volume | 670 (126-1882) | 1123 (218-8316) | 0.29 |
| ECST area CT | | | |
| WMH | <50 | 50-70 | P |
| Number | 34 (9-66) | 18 (4-75) | 0.22 |
| Volume | 1048 (217-3246) | 615 (99-6091) | 0.43 |
| NASCET area CT | | | |
| WMH | <50 | 50-70 | P |
| Number | 28 (7-70) | 27 (11-130) | 0.52 |
| Volume | 677 (111-3798) | 1123 (336-10024) | 0.38 |

SUPPLEMENTARY TABLE 4. Explored correlations between WMH burden and continuous characteristics of the main plaque.

ECST diam (echo), degree of stenosis evaluated with the European Carotid Surgery Trial method on echographic images; ECST area (CTA), degree of stenosis evaluated with the European Carotid Surgery Trial method on computed tomography angiography images; NASCET area (CTA), degree of stenosis evaluated with the North American Symptomatic Carotid Endarterectomy Trial method on computed tomography angiography images.

|  | Number of WMH | | Volume of WMH | |
| --- | --- | --- | --- | --- |
| Characteristic | r | p | r | p |
| ECST diam (echo) | -0.08 | 0.56 | -0.01 | 0.92 |
| ECST area (CTA) | -0.04 | 0.78 | 0.05 | 0.73 |
| NASCET area (CTA) | 0.135 | 0.29 | 0.156 | 0.23 |
| Plaque length | 0.035 | 0.99 | 0.012 | 0.50 |
| Plaque volume | -0.207 | 0.11 | -0.161 | 0.21 |
| Plaque density | 0.126 | 0.34 | 0.131 | 0.32 |
| Positive remodeling | 0.003 | 0.98 | -0.055 | 0.67 |
|  | | | | |

SUPPLEMENTARY TABLE 5. Exclusion criteria for the IMPLAC study

CTA, computed tomography angiography; eGFR, estimated glomerular filtration rate by Cockcroft-Gault equation; PM, pace-maker; ICD, implanted cardioverter defibrillator; PFO, patent foramen ovale; CNS, central nervous system.

| Exclusion criteria for the IMPLAC study |
| --- |
| Age <18 or >85 years |
| Contraindications to CTA (eGFR<60 mL/min; history of allergic reaction to iodinated contrast media) |
| Pregnancy or child-bearing potential |
| Specific contraindication to MRI:   - Claustrophobia - Sickle cell anemia - Systemic mastocytosis - Implanted cardiac devices (PM, ICD) - Vascular clips - Vertebral distractors - Infusion pumps - Neurostimulators - Liquor derivations - Any device which could be dispositioned in the presence of a strong magnetic field |
| Dementia |
| Life expectancy less than study follow up (18 months) |
| History of drug abuse, alcohol abuse or any psychiatric or social condition which may contraindicate the participation to a clinical study |
| Vertebral artery occlusion |
| Previous revascularization of the carotid artery |
| Current anti-coagulation |
| Atrial fibrillation not necessitating anticoagulation |
| Known PFO necessitating anti-platelet treatment |
| Previous cerebrovascular accidents |
| Previous infections to the CNS |
| Previous surgery to the CNS |
| History of anoxic damage to the CNS |
| Previous cardiac surgery or positioning of intracardiac devices (excluded coronary stents) |
| History of autoimmune vasculitis |

SUPPLEMENTARY REFERENCES

1 Mancia, G. *et al.* 2013 ESH/ESC guidelines for the management of arterial hypertension: the Task Force for the Management of Arterial Hypertension of the European Society of Hypertension (ESH) and of the European Society of Cardiology (ESC). *European heart journal* 34, 2159-2219, doi:10.1093/eurheartj/eht151 (2013).

2 Calhoun, D. A. *et al.* Resistant hypertension: diagnosis, evaluation, and treatment: a scientific statement from the American Heart Association Professional Education Committee of the Council for High Blood Pressure Research. *Circulation* 117, e510-526, doi:10.1161/CIRCULATIONAHA.108.189141 (2008).

3 Task Force on diabetes, p.-d. *et al.* ESC guidelines on diabetes, pre-diabetes, and cardiovascular diseases developed in collaboration with the EASD - summary. *Diabetes & vascular disease research* 11, 133-173, doi:10.1177/1479164114525548 (2014).

4 European Association for Cardiovascular, P. *et al.* ESC/EAS Guidelines for the management of dyslipidaemias: the Task Force for the management of dyslipidaemias of the European Society of Cardiology (ESC) and the European Atherosclerosis Society (EAS). *European heart journal* 32, 1769-1818, doi:10.1093/eurheartj/ehr158 (2011).

5 D'Agostino, R. B., Sr., Pencina, M. J., Massaro, J. M. & Coady, S. Cardiovascular Disease Risk Assessment: Insights from Framingham. *Global heart* 8, 11-23, doi:10.1016/j.gheart.2013.01.001 (2013).

6 Bots, M. L., Hoes, A. W., Koudstaal, P. J., Hofman, A. & Grobbee, D. E. Common carotid intima-media thickness and risk of stroke and myocardial infarction: the Rotterdam Study. *Circulation* 96, 1432-1437 (1997).

7 Hollander, M. *et al.* Comparison between measures of atherosclerosis and risk of stroke: the Rotterdam Study. *Stroke; a journal of cerebral circulation* 34, 2367-2372, doi:10.1161/01.STR.0000091393.32060.0E (2003).

8 Grant, E. *et al.* Carotid Artery Stenosis: Gary-scale and Doppler US Diagnosis -Society of Radiologist in Ultrasound Consensus Conference. *Radiology* 229, 340-346 (2003).

9 Randomised trial of endarterectomy for recently symptomatic carotid stenosis: final results of the MRC European Carotid Surgery Trial (ECST). *Lancet (London, England)* 351, 1379-1387 (1998).

10 Mallett, C., House, A. A., Spence, J. D., Fenster, A. & Parraga, G. Longitudinal ultrasound evaluation of carotid atherosclerosis in one, two and three dimensions. *Ultrasound in medicine & biology* 35, 367-375, doi:10.1016/j.ultrasmedbio.2008.09.008 (2009).

11 Pourcelot, L. *et al.* Ultrasound characterization and quantification of carotid atherosclerosis lesions. *Minerva cardioangiologica* 47, 15-24 (1999).
